# Label-free quantitative mass spectrometry analysis of differential protein expression in the developing cochlear sensory epithelium

**DOI:** 10.1186/s12953-018-0144-6

**Published:** 2018-08-07

**Authors:** Lancia N. F. Darville, Bernd H. A. Sokolowski

**Affiliations:** Morsani College of Medicine, Department of Otolaryngology-HNS, University of South Florida, 12901 Bruce B. Downs Blvd, Tampa, FL 33612 USA

**Keywords:** Cochlea, Development, Networks, Quantitative mass spectrometry, Proteomics, Sensory epithelium, Hair cells

## Abstract

**Background:**

The sensory epithelium of the inner ear converts the mechanical energy of sound to electro-chemical energy recognized by the central nervous system. This process is mediated by receptor cells known as hair cells that express proteins in a timely fashion with the onset of hearing.

**Methods:**

The proteomes of 3, 14, and 30 day-old mice cochlear sensory epithelia were revealed, using label-free quantitative mass spectrometry (LTQ-Orbitrap). Statistical analysis using a one-way ANOVA followed by Bonferroni’s *post-hoc* test was used to show significant differences in protein expression. Ingenuity Pathway Analysis was used to observe networks of differentially expressed proteins, their biological processes, and associated diseases, while Cytoscape software was used to determine putative interactions with select biomarker proteins. These candidate biomarkers were further verified using Western blotting, while coimmunoprecipitation was used to verify putative partners determined using bioinformatics.

**Results:**

We show that a comparison across all three proteomes shows that there are 447 differentially expressed proteins, with 387 differentially expressed between postnatal day 3 and 30. Ingenuity Pathway Analysis revealed ~ 62% of postnatal day 3 downregulated proteins are involved in neurological diseases. Several proteins are expressed exclusively on P3, including Parvin α, Drebrin1 (Drb1), Secreted protein acidic and cysteine rich (SPARC), Transmembrane emp24 domain-containing protein 10 (Tmed10). Coimmunoprecipitations showed that Parvin and SPARC interact with integrin-linked protein kinase and the large conductance calcium-activated potassium channel, respectively.

**Conclusions:**

Quantitative mass spectrometry revealed the identification of numerous differentially regulated proteins over three days of postnatal development. These data provide insights into functional pathways regulating normal sensory and supporting cell development in the cochlea that include potential biomarkers. Interacting partners of two of these markers suggest the importance of these complexes in regulating cellular structure and synapse development.

**Electronic supplementary material:**

The online version of this article (10.1186/s12953-018-0144-6) contains supplementary material, which is available to authorized users.

## Background

The cochlear sensory epithelium contains specialized receptors known as hair cells, which are responsible for transducing incoming mechanical signals for processing by the brain [1]. Damage to hair cells can lead to hearing loss or impairment in both humans and mouse [2]. The perception and processing of sound are dependent on the expression of many proteins of which many are regulated during the onset of hearing. As structural and functional development continues, hearing sensitivity increases on postnatal days 12–14 (P12-P14) [3]. A number of studies have investigated gene expression in the inner ear and its age-related changes [4–7]. There have been far fewer studies of the inner ear performed at the proteome level [8–14], and even fewer that have explored protein pathways [15, 16].

Proteomics can provide insights into understanding complex biological systems by analyzing expression, function, modifications, and interactions. To determine the relative differences in protein expression in a cell or tissue, at a given time or under a particular condition, one can use quantitative MS-based proteomics that uses labeled or label-free proteins [17]. Commonly used labeling techniques include isobaric tags for relative and absolute quantitation (iTRAQ) [18], isotope-coded affinity tags (ICAT) [19], tandem mass tags (TMT) [20] and stable isotope labeling by amino acids in cell culture (SILAC) [21]. These techniques are relatively accurate but have limitations, since they are costly, limited by the number of samples that can be analyzed per experiment, and have incomplete labeling efficiencies [17, 22]. In label-free quantitation, two approaches can be used, (i) measurement of the chromatographic elution peak area [23] or (ii) spectral counting [24]. Measurement of peak area involves calculating and comparing the mean intensity of peak areas for all peptides from each protein in the biological sample [25]. In contrast, spectral counting is based on the number of MS/MS spectra generated from a protein. The more abundant the protein in the biological sample, the more peptides will be selected for fragmentation [17]. Both techniques are useful for quantifying differences between proteins, especially for proteins of low abundance [26].

In the present study, we used label-free quantitative proteomics to identify proteins that are differentially expressed in the cochlear sensory epithelium of the mouse between three different ages. We collected protein samples from the cochlear sensory epithelia of P3, P14, and P30 mice, performed multi-digestion procedures, separated peptides using SCX chromatography and analyzed peptides with nano RP-LC-MS/MS. Differential protein expression was determined using spectral counting and an ANOVA was used to determine significant differences in protein expression. Functions for differentially expressed proteins and putative protein partners for select biomarkers were explored using bioinformatics. For verification, select proteins that were differentially expressed were analyzed using immunoblotting and putative partners, determined via bioinformatics, were verified using coIP. This is the first study to identify regulated proteins from the mouse cochlear sensory epithelia before, during, and after the onset of hearing.

## Methods

### Protein extraction from sensory epithelia

The experiments described herein were approved by the University of South Florida Institutional Animal Care and Use Committee, as set forth under the guidelines of the National Institutes of Health. To obtain enough protein for analysis, cochleae were isolated from 16 P3, P14, and P30 CBA/J mice. In addition, three biological replicates were prepared for each age group. All dissections were accomplished in cooled PBS, while maintaining the dissecting dish on ice. To extract a cochlea, the tympanic bulla was excised after which the bone, ligament, and stria vascularis was removed, isolating the sensory epithelium along with the modiolus. The modiolus was kept intact, initially, since this method provided an intact extraction of the sensory epithelium. Three biological replicates from each age group were prepared for LC-MS/MS analysis. In each experiment, the cochlear sensory epithelium was washed gently 3× in 1X PBS, centrifuged for 3 min at 1000 *g*, and the supernatant removed. These washes allowed for the removal of the modiolus prior to detergent treatment. Cochlear sensory epithelia were sonicated in lysis buffer containing 4% (*w*/*v*) SDS, 100 mM Tris-HCl, pH 8.0, 120 mM NaCl, 50 mM NaF, 5 mM EDTA, 500 μg/mL AEBSF, 10 μg/mL leupeptin, 100 μg/mL pepstatin, 2 μg/mL aprotinin, and 1 mg/mL microcystin using a sonic dismembrator (Model 100; Thermo Fisher). The extract was incubated on ice for 30 min, then heated at 95 °C for 4 min, followed by centrifugation at 16000 *g* at 4 °C for 15 min. The supernatant was removed and the pellet extracted in lysis buffer. Both lysates were combined, then centrifuged at 20800 *g* at 4 °C for 60 min. The supernatant was retained for digestion and analysis.

### Multi-FASP digestion

The protein supernatant from above was directly added to a 30 K spin filter and mixed with 200 μL of 8 M urea in Tris-HCl and centrifuged at 14000 *g* for 15 min. The concentrate was diluted with 200 μL of urea solution and centrifuged at 14000 *g* for 15 min. Then, 10 μL of 10 X IAA in urea solution was added to the concentrate in the filter and vortexed for 1 min. The spin filter was incubated for 20 min at RT in the dark followed by centrifugation at 14000 *g* for 10 min. To the concentrate on the filter, 100 μL of urea solution was added and centrifuged at 14000 *g* for 15 min then repeated 2X. There was 100 μL of 100 mM ABC solution added to the spin filter and centrifuged at 14000 *g* for 10 min then repeated 2X. Then, 0.1 μg/μL of LysC was added 1:100 and incubated O/N at 30 °C. Following incubation, 40 μL of 100 mM ABC solution was added and centrifuged at 14000 *g* for 10 min and repeated 1X to increase peptide yield. Finally, 50 μL of 0.5 M NaCl solution was added to the spin filter and centrifuged at 14000 x g for 10 min.

Following the first digestion, spin filters were washed with 40 μL of urea followed with 2X washes of 40 μL of ddH_2_O, then, 3X washes with 100 μL of 50 mM ABC solution, followed by adding 0.1 μg/μL of trypsin in 1:100 and incubating at 37 °C O/N. Peptides were eluted, acidified with trifluoroacetic acid (TFA), and desalted on a C_18_ MacroSpin column (The Nest Group, Southboro, MA). The concentration of the peptides was determined using a microplate colorimetric assay (BioRad).

### Cation exchange chromatography

Peptides were separated off-line on a 200 × 2.1 mm, 5 μm SCX column (Polysulfoethyl A, The Nest Group) using a gradient of 2–40% B over 50 min with a flow rate of 250 μL/min. Solvent A was 5 mM ammonium formate, pH 3.0 in 25% acetonitrile and 75% ddH_2_O. Solvent B was 500 mM ammonium formate, pH 6.0 in 25% acetonitrile and 75% ddH_2_O. The separation was monitored at 280 nm followed by collecting fractions every 4 min. Fractions were dried using a vacuum centrifuge and resuspended in 15 μL of 0.1% FA for MS analysis.

### LC-MS/MS

Each SCX fraction was analyzed by nano LC-MS/MS. Prior to separation, 5 μL of each peptide fraction was injected onto a 100 μm × 25 mm sample trap (New Objective, Woburn, MA) to remove salts and contaminants. Peptide separation was performed on a 75 μm × 10 cm C_18_ column (New Objective, Woburn, MA) using a gradient from 98% solvent A (95% ddH_2_O and 5% acetonitrile containing 0.1% FA) and 2% solvent B (80% acetonitrile and 20% ddH_2_O containing 0.1% FA) to 40% solvent B over 180 min with a flow rate of 300 nL/min on an Eksigent nanoLC (Thermo Scientific Inc.). Mass spectrometry data were collected using an LTQ Orbitrap mass spectrometer (Thermo Scientific Inc.). A DDA “top 10” method was used with an isolation window of 3 around the precursor and 35 normalized collision energy value (NCE). Full MS scans were acquired in the Orbitrap mass analyzer over the m/z 300–1800 range with resolution 60,000 and MS/MS resolution was 7, 500 with a minimal signal of 2.00E + 03. The MS proteomics data are deposited in the ProteomeXchange Consortium (http://proteomecentral.proteomexchange.org) via the PRIDE partner repository [27] with the dataset identifier PXD001973.

### Data analysis

Sequences were assigned using the MASCOT search engine version 2.3 (Matrix Science) against the UniProt database (2012.01) selected for *Mus musculus* (108,308 entries). The parent and fragment ion maximum precursors were set to ±8 ppm and ± 1.2 Da, respectively. The search included a fixed modification of carbamidomethyl of cysteine and variable modifications of oxidation of methionine and protein N-terminal acetylation. A maximum of two missed cleavages were allowed. Scaffold (Version 4.3.2, Proteome Software) was used to validate peptide and protein identifications. Peptide and protein identifications were accepted if they were greater than 95 and 99% probability, respectively, and contained two or more identified peptides. Peptide assignments were also manually verified by inspection of the tandem mass spectra. In addition, a false discovery rate (i.e. false positives) was determined using Scaffold using the empirical method by counting the number of reverse or randomized hits and dividing by the number of forward hits [28, 29]. Proteins were eliminated when identified as a contaminant, such as keratin. Proteins identified in all three replicates were reported. In Scaffold, spectral counts were normalized to the sum of all spectral counts prior to statistical analysis to observe significance. The data were exported from Scaffold and analyzed using a one-way ANOVA followed by the Bonferonni test using Statistica software (Version 12, StatSoft, Inc.). Proteins between age groups were considered significantly different when *p* ≤ 0.05. Spectral counts correlate with protein abundance [24]. Therefore, the mean normalized spectral counts were used to determine fold changes between age groups.

GO information of significantly different proteins was obtained using UniProt [30]. The program provides annotations to proteins in the UniProt Knowledgebase that are controlled vocabulary terms used to describe molecular function, biological process and location of action of a protein in a cell [31]. Furthermore, to observe networks of differentially expressed proteins, their biological processes, and associated diseases, we used the IPA tool (Ingenuity Systems, Redwood City, CA, www.qiagen.com/ingenuity). A right-tailed Fisher’s exact test was used to compare the number of proteins that participate in a given function or pathway relative to the total number of occurrences of these proteins in all biological functions and pathway annotations stored in the IPA knowledge base. The IPA tool also generates a score for each network. The score is derived from a *p*-value and scores of 2 or higher have at least a 99% confidence of not being generated by random chance alone. IPA identifies the most significant diseases and biological functions of the differentially expressed proteins and the top five are categorized and reported based on their *p*-values.

Cytoscape software [32] was used to discover potential interacting proteins for specific protein markers discovered using MS. The databases used include IntAct, Molecular INTeraction database (MINT), Database of Interacting Proteins (DIP), UniProt, BHF-UCL, MatrixDB, and (Interologous Interaction Database (I2D-IMEx). All interacting proteins were filtered to show only mouse proteins in the interactome.

#### Western blot analysis of proteins differentially expressed on P3 and P30

Lysates were prepared from 16 P3 and P30 CBA/J mice cochleae sensory epithelia, as described above. Protein concentrations were determined by DC Protein assay (Bio-Rad) and equal amounts of proteins (3 μg/lane) from P3 and P30 tissues were resolved on Criterion 4–15% Tris-HCl SDS-PAGE gels (Bio-Rad) and transferred onto a nitrocellulose membrane (Amersham Biosciences). Blots were blocked at RT for 1 h in Tris-buffered saline/Tween 20 [50 mM Tris-HCl, pH 7.5, 120 mM NaCl, 0.05% Tween 20] with 4.5% milk and then probed with respective primary antibodies including, anti-Dbn1 at 1:500, anti-Parvin at 1:800, anti-SPARC at 1:1000, and anti-Tmed10 at 1:1000 (all from Proteintech Group) with rocking O/N at 4 °C. Beta-actin was used as a protein loading control using anti-β actin rabbit polyclonal antibody (Abcam) for detection. Before adding secondary antibody, blots were washed 1X with TBS and 2X with 0.05% Tween/TBS. Membranes were then incubated with a donkey anti-rabbit horseradish peroxidase-conjugated secondary antibody at 1:5000 with rocking at RT for 1 h. Secondary antibody was removed and blots washed 1X with TBS, 2X with 0.05% Tween/TBS, and a final wash with TBS. Immunoreactive bands were developed using ECL (Amersham Biosciences) and Magic Mark XP (Invitrogen) was used as the protein standard to estimate relative mobilities.

### Coimmunoprecipitation

Lysates were prepared from 16 P3 CBA/J mice cochleae sensory epithelia. The lysate for each experiment was divided equally into three tubes and diluted ~ 2 fold with lysis buffer containing 20 mM Tris-HCl, pH 7.5, 120 mM NaCl, 1 mM NaF, 2 mM EDTA, 1 mM EGTA, 500 μg/mL AEBSF, 10 μg/mL leupeptin, 100 μg/mL pepstatin, 2 μg/mL aprotinin, and 1 mg/mL microcystin. One tube was used for an IP of the protein itself (positive control), the second was used to coprecipitate a partner, while the third tube was used as a negative control. IPs were performed using the immunocomplex capture method by first adding 5 μg of either anti-Kcnma1 polyclonal antibody (Chemicon; aa 1184–1200 of mouse Kcnma1) or anti-integrin-linked protein kinase polyclonal antibody (Proteintech Group) to two of three tubes containing lysate and then incubating by rocking for 1 h at 4 °C. Negative controls consisted of incubating lysate in the third tube with ChromPure rabbit IgG (Jackson Laboratories). Thirty μL of rec-Protein G Sepharose 4B Beads (Invitrogen) were then added to the three samples and incubated for 1 h at 4 °C. Immunocomplexed beads were washed 1X in PBS, 3X in PBS/0.1% Triton x-100, 1X in PBS, and immunocomplexes recovered by heating at 95 °C for 5 min in Laemmli sample buffer (Sigma-Aldrich). Samples were fractionated on a 7.5% Tris-HCl gel (Bio-Rad) and transferred to a nitrocellulose membrane (Amersham Biosciences). Blots were blocked at RT for 1 h in Tris-buffered saline/Tween 20 [50 mM Tris-HCl, pH 7.5, 120 mM NaCl, 0.05% Tween 20] with 4.5% milk and probed with either anti-SPARC polyclonal antibody at 1:1000 (Proteintech Group) or anti-Parvin polyclonal antibody at 1:800 (Proteintech Group) with rocking O/N at 4 °C to determine the Kcnma1 and Ilk coprecipitates, respectively. The secondary antibody for both consisted of a mouse anti-rabbit light chain at 1:15,000 with rocking at RT for 1 h. Bands were developed using ECL (Amersham Biosciences) and Magic Mark XP (Invitrogen) was used as the standard.

### Reciprocal coimmunoprecipitation

Reciprocal coIPs were performed as described previously in the coIP section, except antibodies to the coprecipitates, SPARC and Parvin were used to coprecipitate Kcnma1 and Ilk, respectively, from P3 lysate. Antibodies and techniques used in the immunocomplex capture method were as before. Blots were probed with BKα polyclonal antibody at 1:400 (Chemicon) and Ilk-polyclonal antibody at 1:500 (Proteintech Group) with rocking O/N at 4 °C, followed with donkey anti-rabbit horseradish peroxidase-conjugated secondary antibody at 1:7500 or with mouse anti-rabbit light chain at 1:15,000 with rocking at RT for 1 h, respectively. Negative controls were as before and positive controls consisted of lysate probed with SPARC and Parvin. Immunoreactive bands were developed as described previously.

## Results

### Protein identification and differential expression

**Fig. 1:**
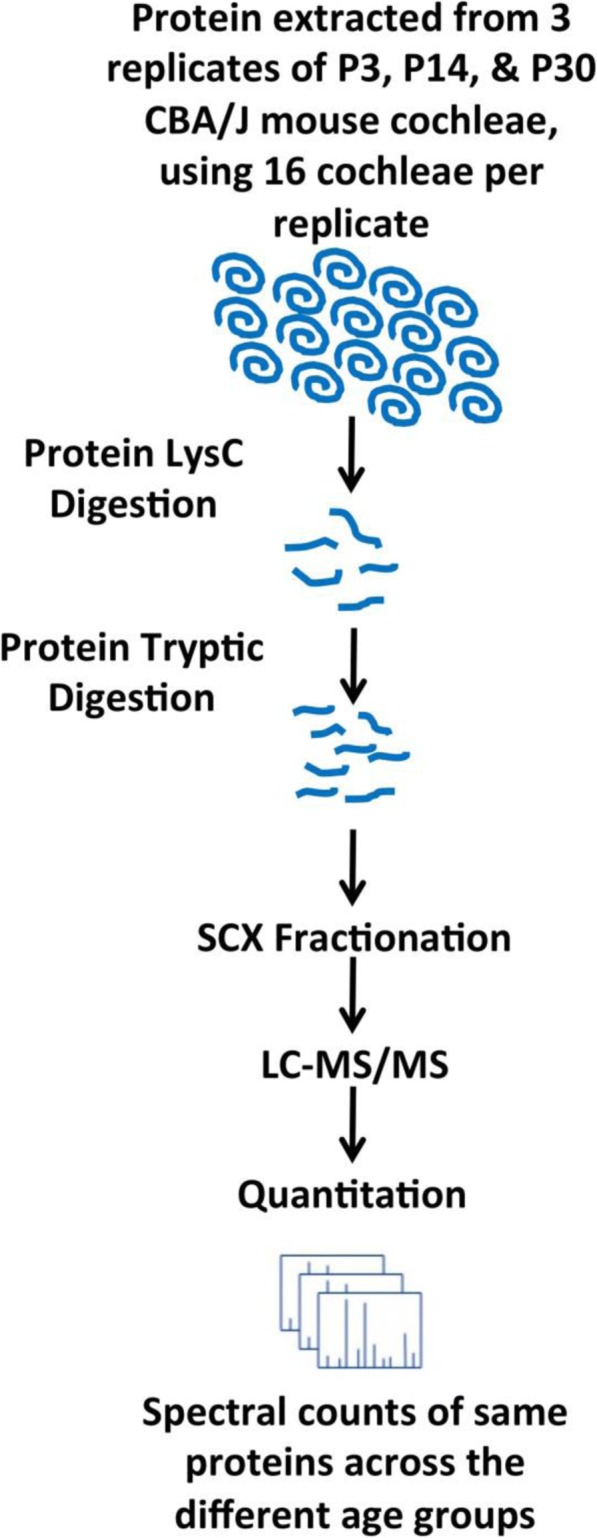
Schematic representation of the protocol used to do label-free quantitative proteomics of P3, P14, and P30 cochlear sensory epithelia of normal hearing CBA/J mice. Cochleae were isolated and the lysate collected and digested using multiple enzymes in the FASP procedure, followed by SCX separation and analysis using LC-MS/MS. Three biological replicates were processed and analyzed for each age group

**Fig. 2:**
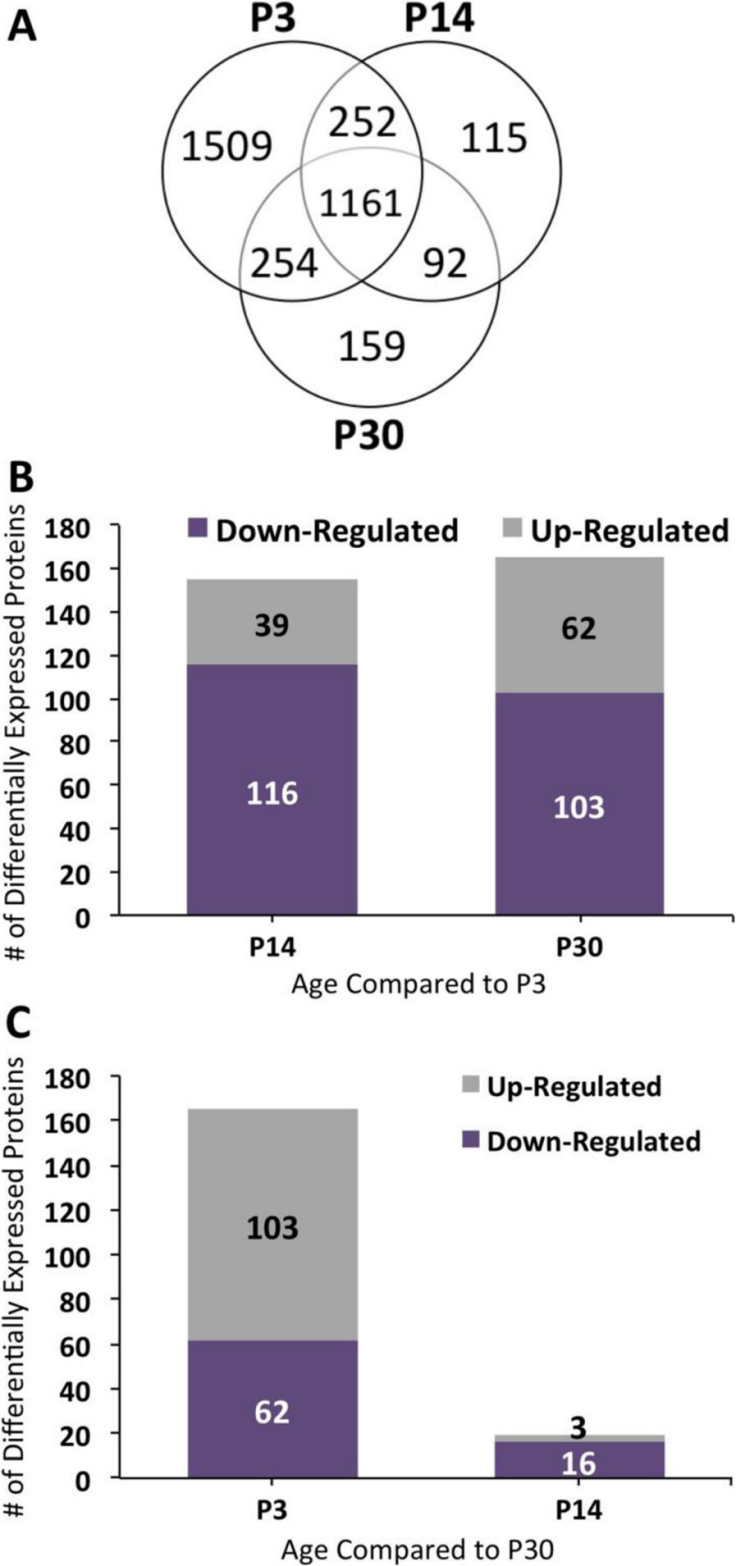
Total proteome and differentially expressed proteins in each age group. **a** Venn diagram showing proteins identified from P3, P14, and P30 proteome. The overlap represents proteins common to these proteomes. **b** Bar graph representing the total number of differentially expressed proteins per age group compared to P30. **c** Bar graph representing the total number of differentially expressed proteins per age group compared to P3

Cochleae from three biological replicates from three different age groups, P3, P14, and P30 were solubilized to extract proteins that were first digested with LysC endoprotease followed by a second digestion with trypsin. Each digest was separated into 14 fractions using SCX and analyzed by nano LC-MS/MS. Spectral counts were used to quantitatively differentiate between proteins among different ages (Fig. 1). A total of 3176, 1620, and 1666 protein were identified on P3, P14, and P30, respectively. All proteins identified from each age group are listed in Additional file 1**:** Table S1. The results in Fig. 2a show that P3 relative to the other ages has the largest number of proteins unique to its age, while there are 1197 proteins common between the three age groups. A one-way ANOVA was conducted to determine differential expression protein candidates on P3, P14, and P30 cochlear sensory epithelium. There was a statistically significant effect of age on protein expression (df = 15, *p* < 0.05; F-values are reported in Additional file 2**:** Table S2). A Bonferonni *post-hoc* test revealed that 447 proteins were significantly different in abundance between the three age groups (p < 0.05). There were 25 proteins differentially expressed between P14 and P30 as compared to 359 and 389 differentially expressed proteins between P3 and P14 and between P3 and P30, respectively (Additional file 2**:** Table S2). Among the differentially expressed proteins, from P3 compared to P14 and P30, were 307 that were common between these groups.

Figure 2b and c show the total number of proteins either up- or downregulated per two age group comparisons. Proteins only present at a particular age were observed separately. We compared the mean normalized spectral counts to identify proteins that were upregulated during development. The data show there are 359 proteins differentially expressed between P3 and P14, and of these, 116 and 39 proteins are up- and downregulated on P3, respectively, with a greater than two-fold change. In comparison, of the 389 proteins differentially expressed between P3 and P30, 103 and 62 are up- and downregulated on P3, respectively, with a greater than two-fold change. The 25 proteins with the largest fold change between P3 and 14 and between P3 and P30 are listed in Tables 1 and 2, respectively. A complete list is in Additional file 3**:** Table S3.

**Table 1:** The 25 selected proteins with upregulated expression on P14 relative to P3. The differentially expressed proteins exhibited the largest fold change

Access. No.	Protein name	Mean normalized spectral counts ± S.D.		Fold change	*P-*value
		P3	P14		
Q61711	Bone sialoprotein 2	0.23 ± 0.56	70.8 ± 37	310	3.68E-04
P19137	Laminin subunit alpha-1	0.20 ± 0.48	26.5 ± 7.4	130	1.01E-04
Q8K482	EMILIN-2	1.31 ± 1.75	81.2 ± 17.0	62	6.88E-08
F6VVY4	Protein Slc25a1	0.16 ± 0.4	5.30 ± 4.38	32	4.18E-02
P27573	Myelin protein P0	4.76 ± 4.46	138 ± 86.8	29	9.51E-03
E9QQ57	Periaxin	5.17 ± 3.47	135 ± 44.2	26	1.42E-03
P70663	SPARC-like protein 1	4.34 ± 3.92	93.5 ± 93.5	22	5.55E-03
O55128	Histone deacetylase complex subunit SAP18	0.59 ± 1.45	10.8 ± 8.15	18	7.39E-03
P07309	Transthyretin	3.47 ± 7.14	47.9 ± 32.13	14	3.39E-02
P29699	Alpha-2-HS-glycoprotein	20.4 ± 20.5	265 ± 78.57	13	1.51E-03
P15105	Glutamine synthetase	1.43 ± 2.27	18.1 ± 12.35	13	3.16E-02
D3Z3Y6	Beta-tectorin	0.74 ± 1.82	8.80 ± 5.3	12	2.05E-02
P16330	2′,3′-cyclic-nucleotide 3′-phosphodiesterase	12.5 ± 5.25	100 ± 33.3	8.0	4.07E-03
P97298	Pigment epithelium-derived factor	4.49 ± 2.62	35.5 ± 28.9	7.9	2.01E-02
P14094	Sodium/potassium-transporting ATPase subunit beta-1	4.49 ± 2.95	32.1 ± 16.8	7.2	3.64E-03
P19246	Neurofilament heavy polypeptide	4.85 ± 5.62	34.1 ± 9.51	7.0	4.70E-04
F8VQ43	Laminin subunit alpha-2	26.9 ± 3.71	165 ± 64.9	6.1	1.39E-04
Q64521	Glycerol-3-phosphate dehydrogenase	2.34 ± 3.09	13.4 ± 4.95	5.7	4.51E-02
P16858	Glyceraldehyde-3-phosphate dehydrogenase	7.87 ± 6.16	41.2 ± 23.2	5.2	6.91E-03
P06745	Glucose-6-phosphate isomerase	4.53 ± 3.8	21.9 ± 13.8	4.8	3.58E-02
Q8BH59	Calcium-binding mitochondrial carrier protein Aralar1	5.50 ± 6.15	25.7 ± 9.56	4.7	1.23E-02
P28654	Decorin	6.25 ± 1.94	26.4 ± 14.2	4.2	2.06E-02
Q9D051	Pyruvate dehydrogenase E1 component subunit beta	3.85 ± 2.63	15.6 ± 9.89	4.1	2.91E-02
Q61245	Collagen alpha-1(XI) chain	14.6 ± 5.7	58.6 ± 33.6	4.0	1.29E-02
Q9JHI5	Isovaleryl-CoA dehydrogenase	4.08 ± 3.84	16.5 ± 11.3	4.0	4.24E-02

**Table 2:** The 25 selected proteins with upregulated expression on P30 relative to P3. These differentially expressed proteins exhibited the largest fold change

Access. No.	Protein name	Mean normalized spectral counts ± S.D.		Fold change	*P-*value
		P3	P30		
P19137	Laminin subunit alpha-1	0.20 ± 0.48	44.0 ± 11.4	220	2.29E-07
Q61711	Bone sialoprotein 2	0.23 ± 0.56	46.3 ± 18.3	200	1.32E-02
P60202	Myelin proteolipid protein	0.33 ± 0.80	59.9 ± 13.0	180	1.96E-06
Q62507	Cochlin	9.79 ± 10.35	780 ± 557	80	5.63E-03
Q8K482	EMILIN-2	1.31 ± 1.75	83.5 ± 14.8	64	4.71E-08
P17879	Heat shock 70 kDa protein 1B	0.31 ± 0.75	14.4 ± 9.16	47	4.26E-03
E9QQ57	Periaxin	5.17 ± 3.47	210 ± 75.9	41	1.27E-05
P07758	Alpha-1-antitrypsin 1–1	0.33 ± 0.81	12.3 ± 11.2	37	4.63E-02
P27573	Myelin protein P0	4.76 ± 4.46	176 ± 74.2	37	1.30E-03
Q80YN3	Breast carcinoma-amplified sequence 1 homolog	2.21 ± 4.14	55.2 ± 33.8	25	4.13E-03
E0CXN5	Glycerol-3-phosphate dehydrogenase	0.31 ± 0.75	6.63 ± 5.35	22	3.94E-02
P15105	Glutamine synthetase	1.43 ± 2.27	26.9 ± 11.7	19	1.39E-03
P51910	Apolipoprotein D	2.23 ± 2.80	39.7 ± 23.6	18	1.53E-02
P03995	Glial fibrillary acidic protein	3.39 ± 7.37	62.5 ± 36.4	18	7.59E-04
P70663	SPARC-like protein 1	4.34 ± 3.92	74.0 ± 33.5	17	7.59E-04
Q8BGR2	Leucine-rich repeat-containing protein 8D	0.55 ± 1.35	9.43 ± 4.07	17	6.29E-04
P62761	Visinin-like protein 1	1.08 ± 1.68	16.6 ± 16.1	15	4.58E-02
P63213	Guanine nucleotide-binding protein subunit gamma-2	1.19 ± 1.86	18.1 ± 9.39	15	2.73E-03
Q9JI59	Junctional adhesion molecule B	0.40 ± 0.97	4.56 ± 3.95	12	3.21E-02
P16330	2′,3′-cyclic-nucleotide 3′-phosphodiesterase	12.5 ± 5.25	145 ± 58	12	8.60E-05
Q62433	Alpha-2-HS-glycoprotein	20.4 ± 20.5	207 ± 145	10	1.25E-02
P29699	Protein NDRG1	2.72 ± 3.36	28.1 ± 15.6	10	2.80E-03
Q62433	Sodium/potassium-transporting ATPase subunit beta-1	4.49 ± 2.95	38.0 ± 12.0	8.5	6.70E-04
P14094	Glutaminase kidney isoform	1.28 ± 1.44	10.7 ± 6.58	8.4	1.24E-02

Based on the two-fold change between P3 and P30, laminin subunit alpha-1, bone sialoprotein 2, and myelin proteolipid are the most highly expressed proteins on P30. In contrast, vimentin, myosin-9, and protein disulfide-isomerase A3 are the most highly expressed proteins on P3. When comparing P3 to P14, there are 4 proteins exclusively expressed on P14, including alpha-2-macroglobulin-P, coagulation factor X, prothrombin, and elongation factor 1-alpha 2. Conversely, there are 200 proteins exclusively expressed on P3. Table 3 lists the top 25 proteins with the largest mean normalized spectral counts for this age, while a complete list is found in Additional file 3**:** Table S3. When comparing P3 to P30, there are nine proteins that exclusively expressed on P30 (Table 4) and 215 proteins exclusively expressed on P3 (Additional file 3**:** Table S3).

**Table 3:** The 25 selected proteins exclusively expressed on P3 relative to P14. These proteins exhibited the highest mean normalized spectral count

Access. No.	Protein name	Mean normalized spectral counts ± S.D.
O54983	Thiomorpholine-carboxylate dehydrogenase	21.7 ± 12.9
Q9Z204	Isoform C1 of Heterogeneous nuclear ribonucleoproteins C1/C2	20.2 ± 6.20
Q6ZQ38	Cullin-associated NEDD8-dissociated protein 1	16.9 ± 2.80
Q9Z1N5	Spliceosome RNA helicase Ddx39b	16.6 ± 7.90
Q99PT1	Rho GDP-dissociation inhibitor 1	15.7 ± 8.60
A2ARV4	Low-density lipoprotein receptor-related protein 2	15.3 ± 4.10
Q9QXS6	Isoform E2 of Drebrin	13.2 ± 9.30
Q9DC51	Guanine nucleotide-binding protein G(k) subunit alpha	11.9 ± 6.50
Q3UQ44	Ras GTPase-activating-like protein IQGAP2	11.7 ± 2.90
E9QP46	Nesprin-2	10.8 ± 4.00
Q00915	Retinol-binding protein 1	10.8 ± 9.10
Q9Z1D1	Eukaryotic translation initiation factor 3 subunit G	10.5 ± 10.9
Q8BVQ9	26S protease regulatory subunit 7	9.3 ± 6.0
Q8BKC5	Importin-5	9.3 ± 7.2
Q9EQH3	Vacuolar protein sorting-associated protein 35	9.0 ± 7.1
Q8VD75	Huntingtin-interacting protein 1	8.4 ± 3.4
Q64511	DNA topoisomerase 2-beta	8.0 ± 4.7
B2RXS4	Plexin-B2	7.9 ± 3.1
Q05186	Reticulocalbin-1	7.9 ± 7.1
Q9CWJ9	Bifunctional purine biosynthesis protein PURH	7.8 ± 5.7
Q8BJ71	Nuclear pore complex protein Nup93	7.8 ± 4.9
P31001	Desmin	7.7 ± 6.0
P97429	Annexin A4	7.5 ± 3.9
Q8BGD9	Eukaryotic translation initiation factor 4B	7.1 ± 6.6

**Table 4:** Proteins exclusively expressed on P30 relative to P3

Access. No.	Protein name	Mean normalized spectral counts ± S.D.
P62631	Elongation factor 1-alpha 2	20.7 ± 9.79
Q6GQT1	Alpha-2-macroglobulin-P	11.5 ± 4.54
P19221	Prothrombin	10.7 ± 6.05
P17809	Acyl-CoA synthetase long-chain family member 6	9.40 ± 8.54
Q5ICG5	Solute carrier family 2, facilitated glucose transporter member 1	8.40 ± 8.77
P22599	Alpha-1-antitrypsin 1–2	5.80 ± 4.88
Q99K67	Alpha-aminoadipic semialdehyde synthase	4.50 ± 3.79
P00920	Carbonic anhydrase 2	4.30 ± 3.82
Q99PU5	Long-chain-fatty-acid--CoA ligase ACSBG1	3.70 ± 3.50

### GO analysis of differentially expressed proteins

**Fig. 3:**
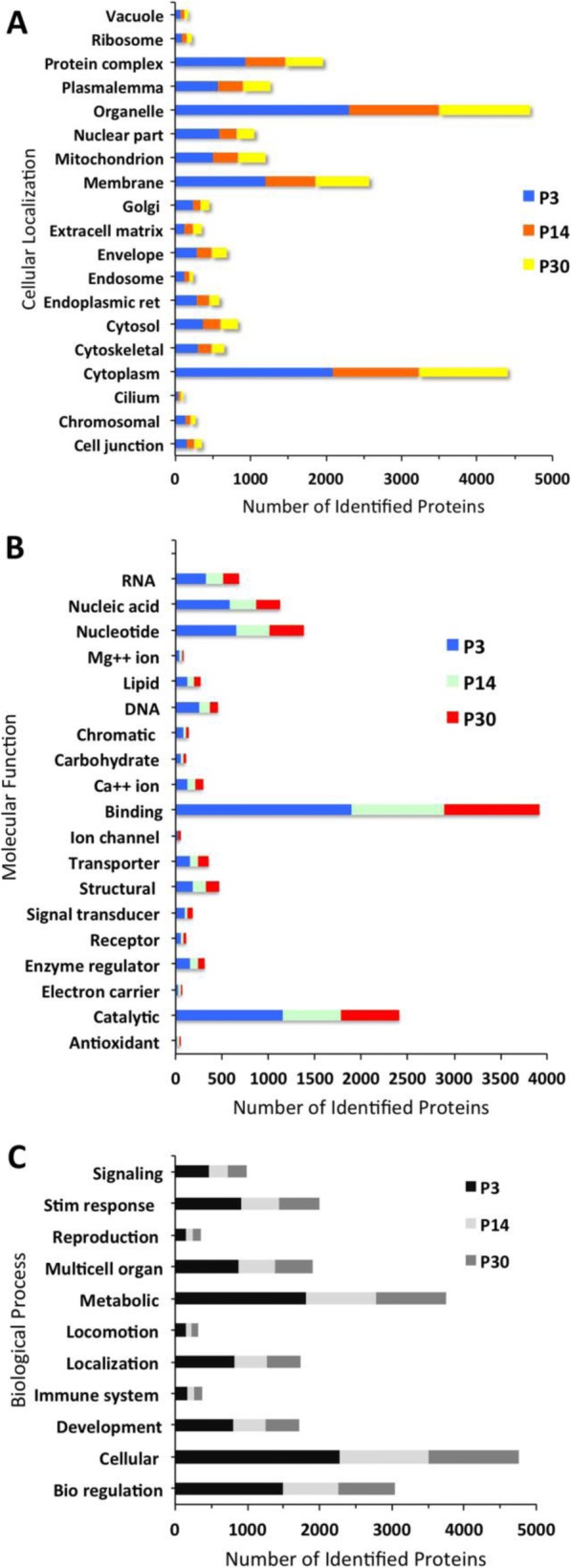
GO classification of proteins identified from P3, P14, and P30 sensory epithelia. Histograms represent (**a**) cellular components, (**b**) molecular function, and (**c**) biological process

The total proteome for P3, P14, and P30 was analyzed using the Gene Ontology database to determine biological processes, cellular localization, and molecular function. All categories were counted non-exclusively, when a protein has more than one category for biological process, cellular localization, and molecular function. Cellular localization analysis of these three proteomes shows a significant number of proteins found in the cytoplasm, organelle, and membrane (Fig. 3a). On P3, P14, and P30, 66, 70, and 71% of proteins are in the cytoplasm, respectively, 73, 74, and 72% of proteins are in organelles, respectively, whereas 38, 41, and 43% of proteins are localized in the membrane, respectively. In contrast, the least number of proteins are localized in the cilium and vacuole. GO shows that on P3, P14, and P30 1, 2, and 2% of proteins are in the cilium, respectively and 2, 3, and 3% of proteins are localized in the vacuole, respectively. Molecular function analysis shows that the most highly expressed proteins on P3, P14, and P30 include binding proteins (60, 62, and 61%, respectively), specifically nucleotide (21, 22, and 22%, respectively) and nucleic acid (18, 18, and 15%, respectively) binding proteins as well as proteins involved in catalytic activity (36, 38, and 38%, respectively) (Fig. 3b). The GO analysis for biological processes shows that the most highly expressed proteins on P3, P14, and P30 are involved in cellular processes (72, 76, and 76%, respectively), metabolic processes (57, 60, and 58%, respectively), and biological regulation (47, 48, and 47%, respectively) (Fig. 3c).

**Fig. 4:**
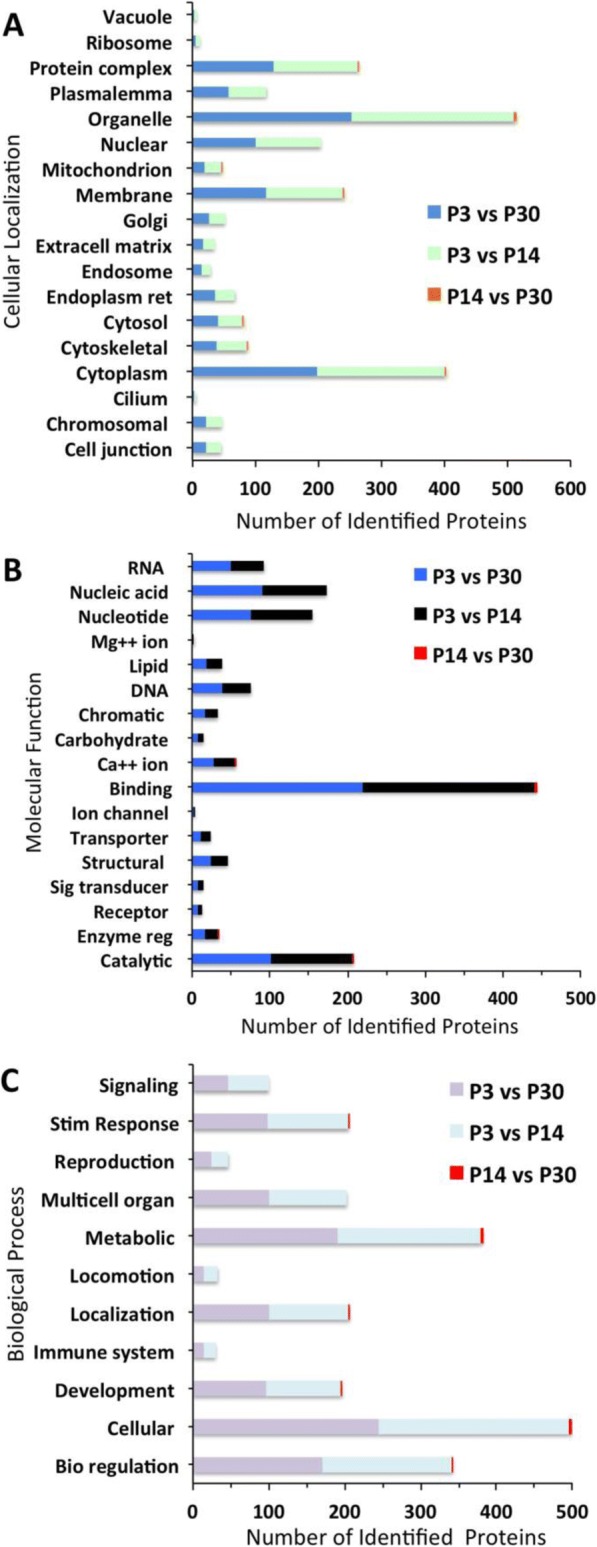
GO classification of proteins significantly upregulated on P3 compared to P14 or P30 and P14 compared to P30. Histograms represent (**a**) cellular components, (**b**) molecular function, and (**c**) biological process

**Fig. 5:**
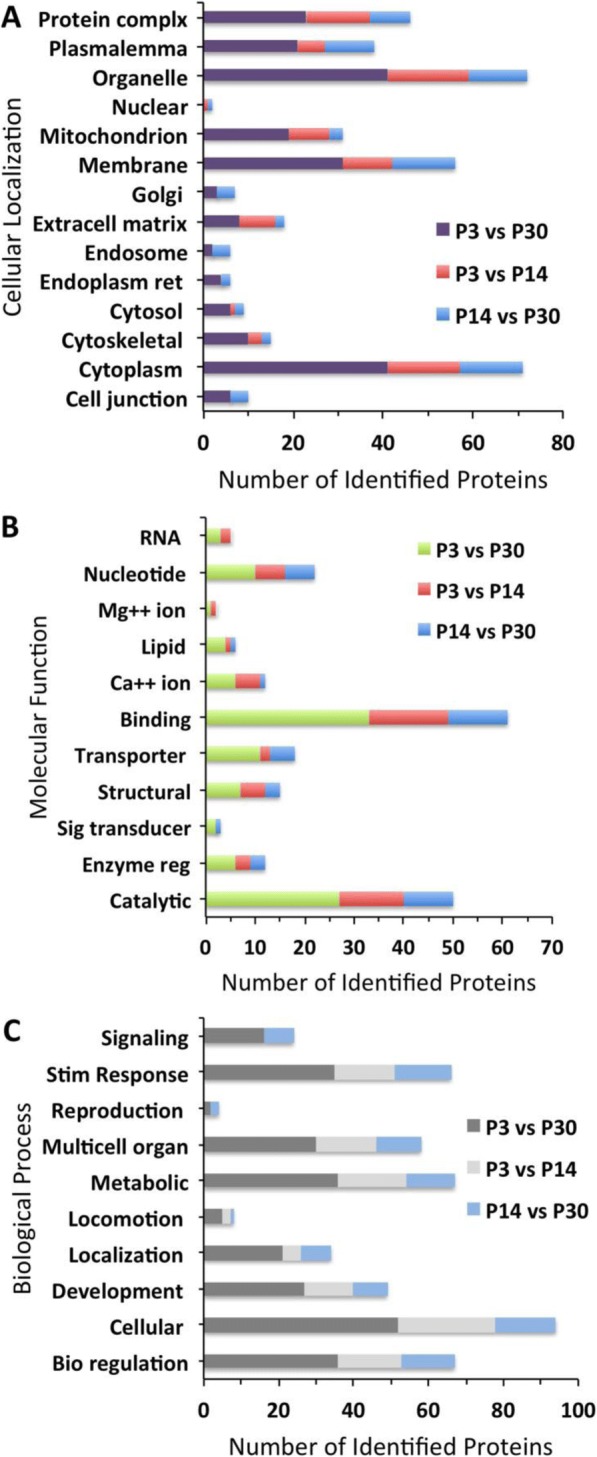
GO classification of proteins significantly downregulated on P3 compared to P14 or P30 and P14 compared to P30. Histograms represent (**a**) cellular components, (**b**) molecular function, and (**c**) biological process

From the 447 differentially expressed proteins, the GO annotations for cellular localization (Fig. 4a), molecular function (Fig. 4b), and biological process (Fig. 4c) of upregulated proteins on P3 relative to P14 and P30 show similar trends. Proteins located in organelles (72 and 65%, respectively), cytoplasm (56 and 51%, respectively), and membrane (34 and 30%, respectively) are the most highly expressed, whereas, as before, cilium (1 and 1%, respectively), vacuole (1 and 1%, respectively), and ribosome (2 and 2%, respectively) are the most lowly expressed. When observing up and downregulated proteins on P3 relative to P14 and P30, these proteins follow a similar trend of expression relative to cellular localization (Fig. 5a), molecular function (Fig. 5b), and biological process (Fig. 5c). In addition, there are no downregulated proteins on P3 relative to P14 involved in signaling or reproduction. The GO analysis for cellular localization, molecular function, and biological process of proteins up- and downregulated on P14 relative to P30 follows a similar trend when compared to the other age groups. However, GO analysis shows that downregulated proteins for cellular localization, molecular function, and biological process are significantly higher on P14 relative to P30. When observing molecular function of the most highly expressed proteins, 48% are involved in binding, 40% catalytic activity and 20% transporter activity. Among the downregulated proteins between all age groups, there are no proteins involved in immune system processes.

### Functional analysis of P3 vs P14 and P3 vs P30

To determine whether there were any significant changes between P3 and P14 developing sensory epithelia, we used IPA to gain insights into function. All IPA analyses are found in Additional file 4**:** Tables S4-S11. An analysis of proteins upregulated on P3 (Additional file 4**:** Table S4) found that, within the physiological system development and function category, 22% associate with tissue development, while 35% associate with organismal survival. Within the disease and disorders function category, many of the proteins associate with hereditary and developmental disorders. A similar IPA analysis of P3 downregulated proteins (Additional file 4**:** Table S5) shows that a majority associate again with tissue development, whereas 62% associate with neurological disease in the disease and disorders category. IPA analyses determining the function of proteins exclusively expressed on P3 and P14 (Additional file 4**:** Tables S6 and S7) show that survival and embryonic/nervous system development proteins (35 and 33%, respectively) are important on P3. Similarly, 75 and 100% of proteins exclusively expressed on P14 associate with tissue morphology and tissue development, respectively.

IPA was utilized also to understand the functions of exclusively and differentially expressed proteins between P3 and P30 sensory epithelia. Analyses show that 27% of proteins upregulated on P3 associate with hereditary disorders (Additional file 4**:** Table S8). In contrast, proteins downregulated on P3, are significantly involved in tissue morphology, nervous system and tissue development, while 65% of P3 downregulated proteins associate with neurological diseases (Additional file 4**:** Table S9). Of additional interest were proteins exclusively expressed in the respective proteomes of P3 and P30 (Additional file 4**:** Tables S10, S11). There were 11 and 24% of proteins exclusively expressed on P3 that are relevant to tissue morphology and renal and urological disease, respectively. On P30, proteins predominate that are relevant to tissue morphology and development. Proteins exclusively expressed on P30 associate with neurological disease and hereditary disorder.

### Validation of potential candidate protein markers by western blot, coIP, and reciprocal coIP

**Fig. 6:**
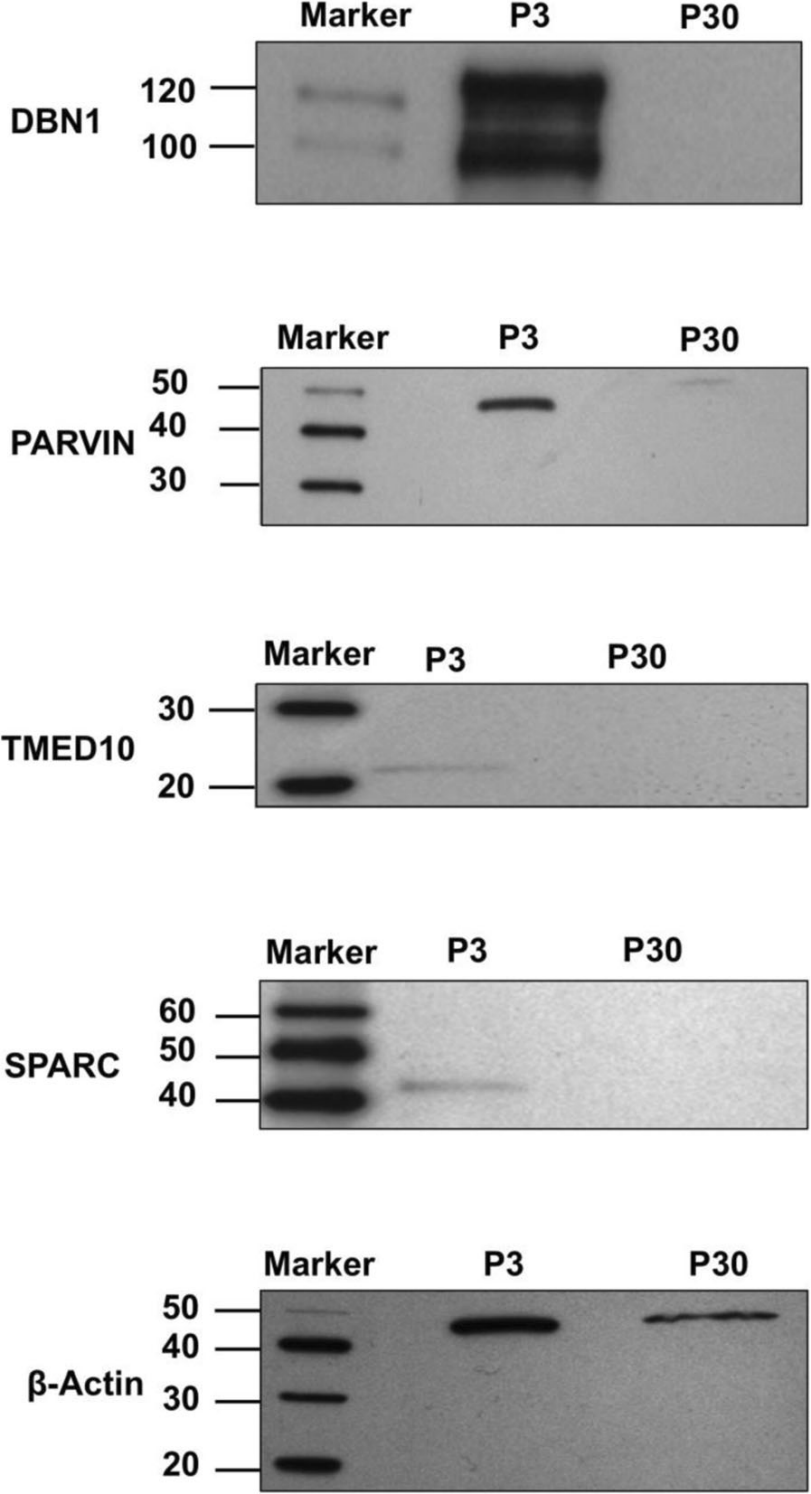
Western blot analysis of proteins predicted as exclusively expressed on P3. Equal amounts of protein lysate from P3 and P30 were loaded onto a gel. All proteins showed expression on P3 and not on P30, except Parvin, which was expressed as a faint peptide species of ~ 50 kDa on P30. The panels show exclusive expression of Dbn1 (100 & 120 kDa), Parvin (42 kDa), Tmed10 (21 kDa), and SPARC (43 kDa) on P3. β-actin was used as a loading control

Western blots were used to validate the expression of selected proteins detected by MS. Candidate proteins were selected based on their exclusive expression between age groups (e.g., P3 relative to P14 and/or P30) as well as by their different functions and newly identified expression in the cochlea. Four potential protein markers identified by MS analysis as exclusively expressed on P3, Parvin, Dbn1, Tmed10, and SPARC, were chosen for verification by comparing expression at P3 to P30. Equal amounts of protein lysate were used from P3 and P30. The results verify the MS findings, since the panels show exclusive expression of Dbn1 (100 & 120 kDa), Parvin (42 kDa), Tmed (21 kDa), and SPARC (43 kDa) on P3 but not on P30 (Fig. 6), except for Parvin. A lowly expressed peptide species of ~ 50 kDa was found, suggesting a modified form of Parvin.

**Fig. 7:**
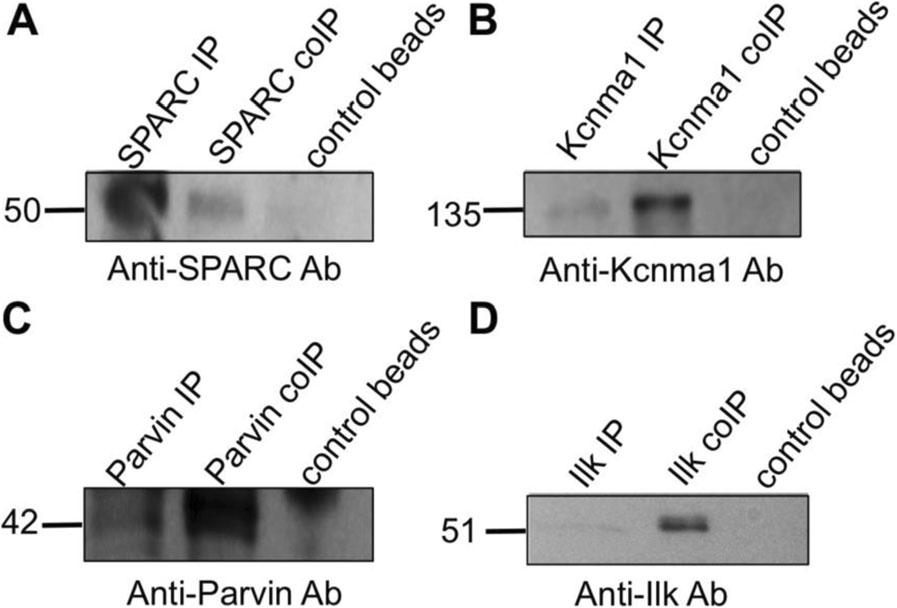
CoIP of putative partners to two proteins exclusively expressed on P3. **a** Kcnma1 coprecipitates SPARC, while (**b**) SPARC coprecipitates Kcnma1 as demonstrated by peptide species of 50 and 135 kDa, respectively. **c** Ilk coprecipitates Parvin, while (**d**) Parvin coprecipitates Ilk as demonstrated by peptides species of 42 and 51 kDa, respectively. Positive control consists of an IP of the protein itself, whereas the negative control consists of pre-incubating lysate with ChromPure rabbit IgG prior to adding beads. The antibody with which each blot was probed appears at the bottom of each panel

Cytoscape analysis of two potential biomarker proteins that were exclusively expressed on P3, Parvin and SPARC, revealed potential binding partners relevant to their function (data not shown). Parvin was found to putatively interact with Ilk, whereas SPARC was found to associate with the Kcnma1, also known as the large conductance calcium-activated potassium channel, or BK. CoIP and reciprocal coIP was used to verify these putative protein-protein interactions. The immunocomplex capture method was used for the coIP and reciprocal coIP of Parvin with Ilk and SPARC with Kcnma1. Kcnma1 coprecipitated SPARC as demonstrated by a peptide species of 50 kDa and SPARC coprecipitated BK as demonstrated by a peptide species of 135 kDa (Fig. 7a and b). Ilk coprecipitated Parvin as demonstrated by a peptide species of 42 kDa and Parvin coprecipitated the lowly expressed Ilk, as demonstrated by a peptide species of ~ 51 kDa (Fig. 7c and d**)**. Negative controls consisted of lysate mixed with IgG-coated beads, resulting in no immunoreactive band. Positive controls consisted of an IP of the protein itself.

## Discussion

The application of MS-based label-free quantitative proteomics resulted in the identification of 447 differentially expressed proteins in the developing mouse cochlear sensory epithelium at P3, P14, and P30. Proteins such as SPARC and unconventional myosin-VI (Myo6), known to be associated with cochlear development, hearing, and deafness, were identified among these proteins. In addition, newly identified proteins recently reported to be associated with the cochlea, such as Parvin [8], exhibited differential protein expression.

### Differential protein expression

The number of up- and downregulated proteins significantly decreased with age when comparing protein expression across the three age groups. These results indicate that there are several structural and functional changes that occur in the P3 mouse cochlear sensory epithelium. These results are consistent with previous reports of structural changes in the aging cochlea [33, 34]. When compared to P3, the number of upregulated proteins significantly increases with age, whereas downregulated proteins show a small decrease. The increase in upregulated protein expression with increased age suggests that many proteins may be involved in physiological and morphological changes that lead to and maintain function. To better understand the significance of these differentially expressed proteins, those related to the development and function of the inner ear were further analyzed using GO and IPA.

Previously, we reported many newly identified proteins in the cochlea [8]. In the present study, we observed that Parvin α and Dbn1, are exclusively expressed on P3 relative to P14, whereas Tmd10 is exclusively expressed on P3 relative to P30. Parvin α is a member of the parvin family of actin-binding proteins and is involved in the reorganization of the actin cytoskeleton, formation of lamellipodia and ciliogenesis [35, 36]. Dbn1 is an actin-binding protein found in the central nervous system that regulates the dendritic spine shape of neurons. This protein plays an important role in the structure-based plasticity of synapses [37], and may thus contribute to early synapse formation, a critical component in controlling the tonotopic organization of these spontaneously active cells [38, 39]. Tmed10 is a member of the p24 family of type I integral-membrane proteins, which are found in the endoplasmic reticulum (ER), the intermediate compartment, and the Golgi apparatus. They are involved in membrane trafficking between the ER and Golgi complex [40]. Hence, this protein may play a role in trafficking membrane proteins to and from stereocilia, thereby maintaining their structure and organization [41].

### Differential expression - inner ear development

Several proteins were expressed differentially that play a role in inner ear development and morphogenesis. These proteins include cadherin-1 (Cdh1), collagen alpha-1 (XI) chain (Col11a1), inactive tyrosine-protein kinase 7 (Ptk7), SPARC, and unconventional myosin-VI (Myo6). SPARC, which is a calcium binding glycoprotein, was exclusively expressed on P3. Interestingly, through bioinformatics and coIP, we found that SPARC interacts with the BK channel. SPARC’s attributes include acting as a trigger for synapse elimination [42], potentially by decreasing the number of docked vesicles in presynaptic active zones [43] through a protein complex that includes integrin [44]. Moreover, integrins can regulate ion channels [45], so that an Integrin/BK/SPARC complex might contribute to the formation of synapses at active zones, since BK is found at both synaptic and extrasynaptic sites [46]. In comparison, Ptk7 was upregulated on P3 relative to P14 and exclusively present on P3 relative to P30. Ptk7 plays a role in cell-cell adhesion, cell migration, cell polarity, proliferation, actin cytoskeleton reorganization, apoptosis, and epithelial tissue organization [47]. Its expression levels on P3, relative to P14 and P30, strongly suggest involvement in development prior to the onset of hearing. In contrast, Cdh1 was present exclusively on P30 relative to P14, and plays a role in cell-cell adhesions, mobility and proliferation of epithelial cells [48]. Additional proteins exclusively expressed on P3 relative to P14 include reticulocalbin-1, plexin-B2, and low-density lipoprotein receptor-related protein 2, which are involved in the development of sensory organs [49] and nervous system [50, 51] and thus may contribute to the development of the cochlear sensory epithelium.

### Differential expression - hearing

A number of hearing-related proteins were expressed differentially, such as collagen alpha-1 (XI) chain, thiomorpholine-carboxylate dehydrogenase, β-tectorin, unconventional myosin-VI, Cdh1, excitatory amino acid transporter 1 (Slc1a3), cochlin, and thiomorpholine-carboxylate dehydrogenase. Thiomorpholine-carboxylate dehydrogenase an oxidoreductase was expressed exclusively on P3, whereas Slc1a3 was expressed exclusively on P30. Proteins that were significantly upregulated on P14 and P30 and exclusively expressed on P30 relative to P3 are of interest, because they may be involved in more mature functions. Laminin subunit alpha-1 and bone sialoprotein 2 (Bsp2) showed the greatest increase on P14 and P30 relative to P3. Laminin, a major component of the basement membrane, is an important regulator of basement membrane assembly and is also involved in cell adhesion, migration, and growth [52]. Increased laminin expression suggests a greater involvement in basement membrane maintenance on P30. Previous work on this structure, using immunohistochemistry, confirms these observations [53]. Bsp2 is a key protein in mineralizing connective tissues [54] The basilar membrane of the inner ear consists of connective tissue composed of cellular and extracellular components [55]. Hence, Bsp2 may play an important role in connective tissue development in the cochlear basilar membrane.

We also identified plasmalemma proteins, such as carbonic anhydrase 2 (CA2) and solute carrier family 2 facilitated glucose transporter member 1 (Slc2a1). These proteins are exclusively expressed on P30 and contribute to auditory function. Carbonic anhydrases are thought to regulate potassium homeostasis and the endocochlear potential in the mammalian cochlea. Previous evidence, using in situ hybridization, showed that CA2 expression within mature mouse inner ear overlapped with Na-K-ATPase in type II and IV otic fibrocytes, suggesting functional relationships [56]. The glucose transporter proteins are members of the major facilitator superfamily of membrane transporters [57]. Neurotransmission between the inner hair cells and their afferent neurons is mediated by glutamate receptors [58]. Glutamate, at low levels, is essential to ensure a high signal-to-noise ratio for afferent neurotransmission and preventing excitotoxic damage to the afferent neurons [59]. High-affinity glutamate transporters are required to rapidly clear synaptic glutamate [60].

### Functional analysis

A number of proteins upregulated, on P3 relative to P14 and P30, associate with hereditary disorders, suggesting these proteins may play a role in genetic hearing loss. These proteins included 14–3-3 epsilon (Ywhae), myosin heavy chain 9 (Myh9), myosin VI (Myo6), and structural maintenance of chromosomes protein 3 (Smc3). The 14–3-3 proteins are a family of regulatory proteins that impact various neurological functions, including neural signaling and development, and neuroprotection [61]. These proteins also play significant regulatory roles in apoptosis, metabolism control, and signal transduction [62] and are associated with many neurodegenerative diseases [61]. Myh9 and Myo6 mutations underlie deafness [63, 64]. Myh9 is expressed in the inner and outer hair cells, spiral ligament and Reissner’s membrane [65]. Myo6 is important for stereocilia development, morphological and functional maturation of the inner hair cell ribbon synapses, and in anchoring the apical hair cell membrane to the cuticular plate [66].

The biological functions predicted for proteins downregulated on P3 relative P14 and P30, as well as proteins exclusively expressed on P14 and P30 relative to P3, are associated with neurological disease as well as tissue development and morphology. This result suggests that these proteins may function in sensorineural hearing loss and that some development and maintenance continues after the onset of hearing. There were three proteins associated with neurological disease as well as tissue development, including ADP/ATP translocase 2 (Slc25a5), 2′,3′-cyclic-nucleotide 3′-phosphodiesterase (Cnp), and sodium/potassium-transporting ATPase subunit beta-1 (Atp1b1). Slc25a5 plays a role in ion transport and Cnp is a membrane-bound protein that serves as a regulator of tubulin polymerization and microtubule distribution [67]. Atp1b1 is a key protein for maintaining cochlear homeostasis. In contrast, the Na+, K + -ATPases take up K+ with high affinity and drive further uptake of K+ via the Na + –2Cl − –K+ cotransporter, thereby maintaining cochlear homeostasis and function [68].

A recently identified protein in the inner ear, actopaxin (parvin α) [8] is associated with organ morphology. This protein is found exclusively on P3 relative to P14 and interacts with Ilk in the P3 cochlea, as confirmed by our coIPs. Interestingly, a recent study suggests that the integrin/pinch1/parvin (IPP) protein complex regulates apico-basal polarity of mammary cells [69]. Hence, parvin’s early expression may contribute to regulating actin organization at the apical and basal poles of cochlear sensory cells.

## Conclusion

We have identified, for the first time, 447 differentially expressed proteins related to the development of P3, P14, and P30 mouse cochlear sensory epithelia by using MS-based label-free quantitative proteomics. Our results show that upregulated proteins increase with age, suggesting they may have a direct involvement in development. During the onset of hearing, at P12–14, proteins related to epithelial and nervous system development, and tissue morphology are the most represented upregulated proteins, suggesting continued development at this age. We also focused on proteins exclusively present in the cochlear sensory epithelium; nine expressed exclusively on P30 relative to P3 and 200 expressed exclusively on P3 relative to P14. Additionally, we reported three proteins expressed exclusively on P3 that were recently identified in the cochlea for the first time. Our bioinformatics approach provided insights on biological functions and interacting partners for select putative biomarkers found on P3, which were verified using immunoblotting and coIP. This study provides the first differentially expressed proteome in the mammalian cochlea at significant developmental stages; before hearing, during the onset of hearing, and when hearing is fully developed. These results provide insights into the function of proteins that are differentially expressed during development and to potential protein biomarkers related to auditory development and loss.

## Additional Files

Additional file 1: **Table S1.** A complete list of proteins from P3, P14, and P30 proteome. (XLSX 193 kb)

Additional file 2: **Table S2.** Statistical analysis results of differentially expressed proteins using a one-way ANOVA and post-hoc test. (XLS 162 kb)

Additional file 3: **Table S3.** A complete list of differentially expressed proteins on P3, P14, and P30. (XLSX 88 kb)

Additional file 4: **Table S4.** Summary of IPA of proteins upregulated on P3 relative P14. **Table S5.** Summary of IPA of proteins downregulated on P3 relative P14. **Table S6.** Summary of IPA of proteins exclusively expressed on P3 relative P14. **Table S7.** Summary of IPA of proteins exclusively expressed on P14 relative to P3. **Table S8.** Summary of IPA of proteins upregulated on P3 relative P30. **Table S9.** Summary of IPA of proteins downregulated on P3 relative P30. **Table S10.** Summary of IPA of exclusively expressed on P3 relative to P30. **Table S11.** Summary of IPA of proteins exclusively expressed on P30 relative to P3. (XLSX 25 kb)

## Abbreviations

ABC: Ammonium bicarbonate
AEBSF: 4-benzenesulfonyl fluoride hydrochloride
ANOVA: Analysis of variance
CNS: Central nervous system
coIP: Coimmunoprecipitation
DTT: Dithiothreitol
ER: Endoplasmic reticulum
FA: Formic acid
FASP: Filter aided sample preparation
GO: Gene Ontology
IAA: Iodoacetamide
ICAT: Isotope-coded affinity tags
IP: Immunoprecipitation
IPA: Ingenuity Pathway Analysis
IPA: Ingenuity Pathway Analysis
iTRAQ: Isobaric tags for relative and absolute quantitation
LysC: Endoproteinase Lys*-*C
MS: Mass spectrometry
MS/MS: Tandem mass spectrometry
nano LC-MS/MS: Nano liquid chromatography-tandem mass spectrometry
O/N: Overnight
P3: Postnatal day 3
PBS: Phosphate buffered saline
RP: Reversed-phase
S.D.: Standard Deviation
SCX: Strong cation exchange
SDS: Sodium dodecyl sulfate
SILAC: Stable isotope labeling by amino acids in cell culture
TFA: Trifluoroacetic acid
